# Implications of Heart Failure Treatment on Atrial Fibrillation Onset: A Retrospective Study

**DOI:** 10.3390/medicina61030414

**Published:** 2025-02-27

**Authors:** Loredana Suhov, Adrian Apostol, Larissa Dăniluc, Lina Haj Ali, Oana Elena Sandu, Carina Bogdan, Minodora Andor

**Affiliations:** 1 Doctoral School, “Victor Babes” University of Medicine and Pharmacy, Eftimie Murgu Sq. No. 2, 300041 Timisoara, Romania; loredana.ogarcin@umft.ro (L.S.); larissa.daniluc@umft.ro (L.D.); lina.haj-ali@umft.ro (L.H.A.); oana.ciolpan@umft.ro (O.E.S.); carina.bogdan@umft.ro (C.B.); 2 Department of Cardiology, Pius Brinzeu Clinical Emergency County Hospital Timisoara, 300736 Timisoara, Romania; 3 Department VII, Internal Medicine II, Discipline of Cardiology, “Victor Babes” University of Medicine and Pharmacy, Eftimie Murgu Sq. No. 2, 300041 Timisoara, Romania; 4 Department V, Internal Medicine I, Discipline of Medical Semiotics II, “Victor Babes” University of Medicine and Pharmacy, 300041 Timisoara, Romania; andor.minodora@umft.ro; 5 Multidisciplinary Heart Research Centre, “Victor Babes” University of Medicine and Pharmacy, 300041 Timisoara, Romania

**Keywords:** atrial fibrillation, heart failure, ejection fraction, SGLT-2 inhibitors

## Abstract

*Background and Objectives*: Atrial fibrillation (AF) is one of the most common supraventricular arrhythmias in the adult population worldwide and it is frequently associated with heart failure (HF). The coexistence of these conditions increases morbidity, mortality and reduces quality of life in these patients. Therefore, it is important to delay the onset of AF in HF patients in order to avoid complications. The study aims to assess whether HF treatment influences AF onset. *Materials and Methods*: This retrospective observational study included 260 patients, 144 patients with heart failure treated with sodium–glucose cotransporter 2 inhibitors (SGLT2i) and 116 patients with heart failure without SGLT2i treatment (control group) hospitalized at least twice in the Cardiology Department of the “Pius Brinzeu” Emergency County Hospital between 2022 and 2024. *Results*: Treatment with SGLT2i was associated with a lower prevalence of atrial fibrillation in patients with heart failure. *Conclusions*: The study highlights the cardiovascular benefits of SGLT2 inhibitors and suggests a potential effect on the onset of AF in heart failure patients.

## 1. Introduction

Atrial fibrillation (AF) is a supraventricular arrhythmia defined as uncoordinated atrial activity and irregular ventricular response. It is the most common arrhythmia in adults and the leading cause of hospitalization in one third of patients with arrhythmias [1]. In 2019, the global AF prevalence was estimated at 59.7 million cases, a number expected to rise in the following years, due to the aging population and improved diagnostics [2,3]. The most important and feared complication of AF is systemic embolism, especially cerebrovascular embolism, which significantly increases mortality risk in patients with atrial fibrillation [3]. Comorbidities such as diabetes, obesity, hypertension, heart failure, sleep apnea and alcohol consumption are risk factors for AF onset and their management can improve patient outcomes or even prevent AF recurrence [2]. The association of any of these risk factors and AF leads to an increase in morbidity and mortality while reducing the quality of life in these patients [3].

Heart failure (HF) is a complex clinical syndrome characterized by impaired filling and the ejection of blood from the ventricles. Depending on the left ventricle ejection fraction (LVEF), HF can be classified as HF with preserved (HFpEF), mildly reduced (HFmrEF) and reduced ejection fraction (HFrEF). The New York Heart Association (NYHA) functional classification is used to determine HF severity. AF is very common in patients with HF, with prevalence rates ranging from 10% in Class I to 50% in Class IV NYHA patients. In patients with preexisting left ventricular dysfunction, new-onset AF can exacerbate heart failure symptoms [1].

In recent years, SGLT2 inhibitors have emerged as effective agents for managing HF, reducing symptoms, recurrent hospitalizations and improving survival and quality of life in HFrEF patients, regardless of diabetes status [4]. These agents act by reducing glucose reabsorption and inducing glycosuria and natriuresis. This results in a reduction in preload and afterload conferring cardioprotective effects [5]. Additionally, they showed a small decrease in systolic blood pressure of 3–6 mmHg secondary to their natriuretic and osmotic diuretic effects [6].

Recent studies have shown that SGLT2 inhibitors may possess anti-arrhythmic properties, potentially delaying arrhythmias through various mechanisms such as reduced myocardial oxidative stress and inflammatory response. Also, an improvement of endothelial dysfunction and cardiac fibrosis and the amelioration of electrophysiological remodeling may be some of the mechanisms of their anti-arrhythmic effects [7,8].

The study aims to evaluate the influence of SGLT2 inhibitor treatment on atrial fibrillation onset in patients with heart failure with reduced and mildly reduced ejection fraction. Findings may highlight a possible new effect of SGLT2 inhibitors, reducing the need for anti-arrhythmic medication while avoiding their associated effects in these patients.

## 2. Materials and Methods

### 2.1. Study Population

This retrospective observational study included 260 patients with heart failure divided into two groups: 144 patients with heart failure treated with SGLT2 inhibitors (SGLT2i group) and 116 patients with heart failure who did not receive SGLT2 inhibitors (control group). Patients enrolled in this study were admitted in the cardiology department at least twice between February 2022 and December 2024, with the second admission occurring 6 to 12 months after the first. The patients were assigned to one of the groups depending on whether they followed SGLT2 inhibitor treatment or not. Data on demographic characteristics, medical history, echocardiographic parameters and electrocardiograms were obtained for both admissions for each patient. Echocardiography was conducted using the Mindray DC-80 X-Insight (MINDRAY, Shenzhen, China) ultrasound systems equipped with a 1–5 MHz transducer. Standard 12-lead electrocardiograms performed with Nihon Kohden ECG-1350K electrocardiograph (NIHON KOHDEN, Tokyo, Japan) were used to detect atrial fibrillation during each admission.

### 2.2. Inclusion and Exclusion Criteria

Patients included in this study were patients admitted to the cardiology department aged 18 years or older, diagnosed with heart failure with reduced (<40%) or mildly reduced ejection fraction (41–49%), with treatment for heart failure including betablockers.

**Figure 1:**
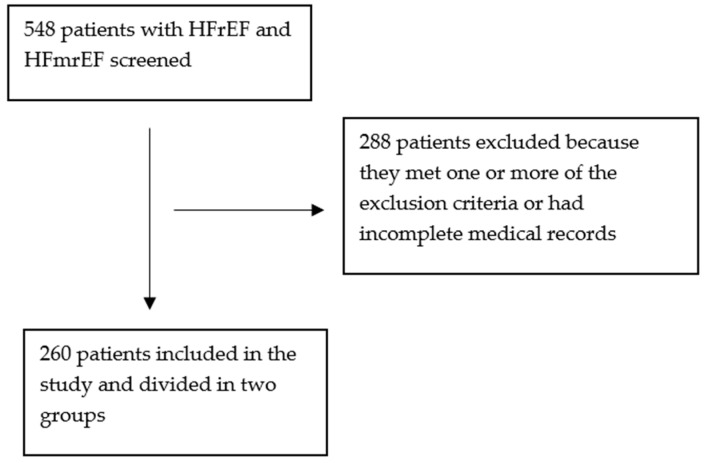
Study population selection process.

The study excluded patients with pre-existing atrial fibrillation and patients without betablocker treatment. The exclusion of these patients ensured a focused evaluation of new-onset atrial fibrillation in heart failure patients without prior AF history. Also, patients treated with anti-arrhythmic drugs other than betablockers, as well as patients with acute infections that could precipitate AF onset, were excluded from this study. Patients with a history of thyroid disorders were excluded from our study due to the potential risk of thyrotoxicosis or treatment overdose that could precipitate AF onset in these subjects. Patients with incomplete or missing medical records were also excluded to ensure the integrity of this study. The study population selection process is illustrated in Figure 1.

### 2.3. Statistical Analysis

Statistical analyses were conducted with the GraphPad Prism 10 software version 10.4.1 and Excel Office 2019. The descriptive statistics employed to summarize the demographic and clinical characteristics of the study population were expressed as means and standard deviations (SDs) for continuous variables (for example, age, ejection fraction, etc.) and percentages for categorical variables (such as sex, smokers and coronary artery disease). Fisher’s exact test was used to assess the percentage differences for categorical variables. We used the independent samples *t*-test to compare the means of continuous variables between the two groups and the paired *t*-test to compare the means of continuous variables in two matched groups. Pearson correlation was applied to assess the relationship between continuous and binary variables. We performed multiple linear regression analyses to adjust for key confounders. A significance level of *p* < 0.05 was set for all tests.

### 2.4. Ethical Consideration

Informed consent for future research was obtained for all participants at the time of admission, in accordance with ethical guidelines. The study obtained approval from the hospital’s ethics board with approval number 23/28 February 2022. All examinations and echocardiographs were performed by experienced cardiologists.

## 3. Results

### 3.1. Study Group Description

The study population consisted of 260 patients with heart failure, 144 patients in the SGLT2i group and 116 patients in the control group. The mean age of the participants was 63.65 ± 11.34 in the group of patients treated with SGLT2 inhibitors and 67.14 ± 11.54 in the control group. In both groups, the majority of participants were men, 72.91% in the SGLT2i group and 68.96% in the control group. The majority of both groups had chronic coronary syndrome, 87.5% in the SGLT2i group and 89.66% in the control group and arterial hypertension, 77.08% in the SGLT2i-treated group and 62.07% in the control group. In the SGLT2i group, 78 people had type 2 diabetes, 81 had chronic kidney disease and 31 suffered from obstructive sleep apnea. In the control group, 56 participants were diabetic, 48 suffered from chronic kidney disease and 14 had obstructive sleep apnea. In total, 64.58% of the SGLT2i group were smokers versus 34.48% of the participants in the control group. Half of the SGLT2i group suffered from obesity while only 24.14% of the people in the control group were obese. The demographic characteristics and comorbidities of the two groups are described in Table 1.

At first admission, the participants of the SGLT2i group had a mean left ventricle end-diastolic volume of 166.9 ± 62.11 mL, a mean of the left ventricle ejection fraction of 32.54 ± 8.98%, a left atrium volume mean of 66.31 ± 21.16 mL and a left atrium diameter mean of 4.38 ± 0.76 cm. The people in the control group had a mean of the left ventricle end-diastolic volume of 155.5 ± 45.67 mL, a mean of the ejection fraction of the left ventricle 32.00 ± 10.31%, a left atrium volume mean of 60.34 ± 15.35 mL and a left atrium diameter mean of 4.13 ± 0.58 cm. There was no statistically significant difference between the means of the left ventricle end-diastolic volume and the ejection fraction of the two groups. The means of the left atrium volume and diameter were different with a statistical significance of 0.009 and 0.003, as stated in Table 2.

Table 3 shows the means of the transthoracic echocardiography parameters at the first and second admission in both groups. In the SGLT2i group, the left ventricle end-diastolic volume mean and the left atrium volume mean were lower at the second admission (*p* = 0.03 and *p* = 0.0003). The left ventricle ejection fraction mean was higher at second admission with a *p* value of 0.01. The left atrium diameter mean was lower at the second admission without statistical significance (*p* = 0.14). The participants in the control group had a higher mean left ventricle end-diastolic volume (*p* = 0.0002), left atrium volume (*p* < 0.0001) and left atrium diameter (*p* = 0.02) at the second admission. The left ventricle ejection fraction mean was lower at the second admission with *p* < 0.0001.

### 3.2. Atrial Fibrillation Onset Analysis

Patients treated with SGLT2 inhibitors had a significantly lower prevalence of atrial fibrillation at the second admission compared to the control group (OR = 4.773, 95% CI: 2.150 to 10.81, *p* value < 0.0001). AF was detected in 6.25% of patients in the SGLT2i group versus 24.14% in the control group (Table 4).

### 3.3. Correlation of Atrial Fibrillation Onset and Comorbidities

Pearson correlation was used to analyze the relationship between the number of comorbidities and the presence of atrial fibrillation (Table 5). In both groups, there is a positive relationship between the number of comorbidities and the onset of atrial fibrillation (r = 0.246, 95% CI = 0.08 to 0.39, *p* = 0.003 in SGLT2i group and r = 0.225, 95% CI = 0.04 to 0.39, *p* = 0.01 in the control group).

### 3.4. Multivariable Regression Analysis for Predictors of New-Onset Atrial Fibrillation

Multiple linear regression analysis was conducted to adjust for potential confounders, including diabetes, hypertension, chronic coronary disease, renal disease, obesity, smoking status and obstructive sleep apnea. SGLT2 inhibitor treatment remained independently associated with a lower risk of new-onset atrial fibrillation (adjusted β = −0.249, 95% CI: −0.338 to −0.161, *p* < 0.0001). Additional significant predictors included renal disease (β = 0.115, *p* = 0.0067) and sleep apnea (β = 0.210, *p* = 0.0002). The model explained 20.7% of the variance (R^2^ = 0.207). Table 6 provides a detailed summary of the results.

## 4. Discussion

This retrospective study demonstrated that treatment with SGLT2 inhibitors in patients with heart failure with reduced and mildly reduced ejection fraction could lower the risk of new-onset atrial fibrillation. Furthermore, the presence of multiple comorbidities such as type 2 diabetes, arterial hypertension, chronic coronary disease, chronic kidney disease, obstructive sleep apnea, obesity and smoking is associated with a higher prevalence of atrial fibrillation in these patients. The observed reduction in left ventricular and left atrial volumes, as well as the left atrial diameter in the SGLT2i group compared to the control group may be linked to a lower prevalence of atrial fibrillation in this group.

Heart failure and atrial fibrillation share common risk factors, and they physiologically increase the effect of each other. HF may induce atrial fibrosis and ionic remodeling, facilitating the development and persistence of AF [9]. Patients with both HF and AF tend to have an increased risk of cardiovascular complications, emphasizing the need for effective therapeutic strategies to improve their quality of life and reduce complications [10].

SGLT2 inhibitors have multiple cardioprotective effects by reducing afterload and preload through arterial vasodilation, natriuresis and diuresis. Other beneficial metabolic effects include upregulation of ketone body, free fatty acids, anti-inflammatory effects, anti-fibrotic effects and the modulation of sympathetic nerve activity on the heart [7,11,12]. Some studies have reported possible reversed cardiac remodeling in patients treated with SGLT2 inhibitors. LV end-diastolic volume and left atrial volume index decreased in diabetic patients treated with empagliflozin for 13 weeks compared to a placebo in a randomized clinical trial conducted by Ersboll et al. [13]. Although the precise mechanisms underlying this effect remain unclear, the inhibition of cardiomyocyte apoptosis has been suggested as one potential explanation [14]. SGLT2 inhibitors may influence cardiac remodeling through different molecular pathways in terms of oxidative stress, energy metabolism, cardiac fibrosis, inflammation, autophagy, apoptosis and ferroptosis, which may be responsible for changes in cardiac structure and function [15].

Treatment with SGLT2 inhibitors has proven effective in reducing HF hospitalizations and cardiovascular mortality in patients with heart failure. Moreover, recent studies have demonstrated benefits in patients with chronic kidney disease, including reduced hospitalizations and a decreased risk of estimated glomerular filtration rate decline, highlighting their cardiac, renal and metabolic effects [5,16]. One meta-analysis evaluated the efficacy of various heart failure medications on cardiovascular death and heart failure hospitalization in different subgroups of patients. Dapagliflozin and empagliflozin were efficient in reducing the main outcome in patients older than 65 years, patients with diabetes mellitus, patients with ischemic HFrEF and patients treated with angiotensin receptor/neprilysin inhibitor. Dapagliflozin was efficient in reducing the primary endpoint in patients with chronic kidney disease while empagliflozin showed greater efficacy in women, pointing up different effectiveness of each drug in a specific patient population [17].

Emerging evidence suggests that SGLT2 inhibitors may possess anti-arrhythmic properties, though the underlying mechanisms are not yet fully elucidated. Indirect mechanisms include reductions in blood pressure, body weight, sympathetic tone and cardiac load in heart failure patients. The direct mechanisms of the SGLT2 inhibitors’ anti-arrhythmic effect appear to act on the myocardium and circulation, ion homeostasis and cardiac electrophysiology [7]. One study evaluated a possible class I anti-arrhythmic effect of SGLT2 inhibitors in human atrial myocytes by inhibiting voltage-gated sodium currents [18]. Similarly, Paasche et al. showed that an increased dose of dapagliflozin reduced atrial cardiomyocyte excitability and could terminate induced AF in porcine models [19].

Despite these promising findings, data on the effect of SGLT2 inhibitors on AF onset in HF patients remain inconclusive. Butt et al. found no reduction in the risk of new-onset AF in patients with HF treated with dapagliflozin [20]. A systematic review and meta-analysis by Li et al. showed that treatment with SGLT2 inhibitors reduced the risk of stroke, heart failure and hospitalization for heart failure, myocardial infarction, unstable angina and cardiovascular mortality but did not reduce the risk of atrial fibrillation [21]. Minguito-Carazo et al. studied the effect of SGLT2 inhibitors on ventricular and atrial arrhythmias in patients with implantable cardiac devices, showing a decrease in the percentage of patients with ventricular arrhythmia but no beneficial effect for atrial arrhythmias [22]. Conversely, some studies have suggested a protective effect against AF. A Mendelian randomization study by Li et al. linked SGLT2 inhibitor treatment to a reduced AF risk [23]. Bonora et al. compared the association of atrial fibrillation with SGLT2 inhibitors and other antidiabetic medications, highlighting a higher frequency of AF in patients treated with diabetes medication other than SGLT2 inhibitors [24]. A Scandinavian cohort study by Engström et al. showed a slightly reduced risk of new-onset AF in patients treated with SGLT2 inhibitors compared to those on glucagon-like peptide 1 receptor agonists [25]. Furthermore, Zelniker et al. found a 19% reduction in AF and atrial flutter events in patients with type 2 diabetes treated with SGLT2 inhibitors [26]. These conflicting findings highlight the need for further real-world data to clarify whether SGLT2 inhibitors confer a protective effect against new-onset AF in HF patients.

This research, however, is subject to several limitations. First, the observational, non-randomized design introduces the potential for selection bias and confounding, as treatment allocation was not randomized but based on clinical decisions. Despite efforts to minimize bias by excluding patients with pre-existing AF, residual confounding may persist due to unmeasured variables. The lack of randomization limits our ability to establish causal relationships between SGLT2 inhibitor treatment and new-onset AF. Future prospective, randomized studies are necessary to validate these findings and establish causality. Second, the lack of serial electrocardiograms and Holter monitoring hindered the ability to quantify atrial fibrillation duration and classify it as paroxysmal or persistent. Also, the reliance on intermittent monitoring increased the likelihood of undetected paroxysmal AF episodes. Third, the time interval between the first and second admissions (6 to 12 months) could over- or underestimate the effect of SGLT2 inhibitors on new-onset atrial fibrillation. A longer follow-up time is required to further study the long-term effects of SGLT-2 inhibitors on AF onset. Finally, this study was conducted in a single hospital, thus the sample size included in the study was small and may limit the universality of our findings. Future studies with larger patient cohorts and more comprehensive monitoring protocols are required to further investigate the potential protective effects of SGLT2 inhibitors on AF onset.

## 5. Conclusions

In conclusion, our study demonstrated a lower prevalence of new-onset atrial fibrillation in patients with heart failure with reduced and mildly reduced ejection fraction treated with SGLT2 inhibitors. Additionally, a reduction in the left ventricle volume and left atrium volume and diameter in the group treated with SGLT2 inhibitors could be linked to a lower percentage of new-onset atrial fibrillation in this group. A positive correlation was observed between the number of comorbidities and atrial fibrillation onset in both groups.

## Figures and Tables

**Table 1:** Characteristics of the study population.

Demographic Characteristics, Comorbidities	SGLT2i Group	Control Group	*p* Value
Age (years)	63.65 ± 11.34	67.14 ± 11.54	0.01 *
Gender:			
Females	39/144 (27.08%)	36/116 (31.03%)	0.49
Males	105/144 (72.92%)	80/116 (68.97%)	0.49
Arterial hypertension	111/144 (77.08%)	72/116 (62.07%)	0.009 **
Chronic coronary syndrome	126/144 (87.5%)	104/116 (89.66%)	0.69
Type 2 diabetes	78/144 (54.17%)	56/116 (48.28%)	0.38
Chronic kidney disease	81/144 (56.25%)	48/116 (41.38%)	0.01 *
Obstructive sleep apnea	31/144 (21.53%)	14/116 (12.07%)	0.04 *
Obesity	72/144 (50%)	28/116 (24.14%)	<0.0001 **
Smokers	93/144 (64.58%)	40/116 (34.48%)	<0.0001 **

Data are presented as number and percentage or mean ± standard deviation; * *p* values < 0.05; ** *p* values < 0.01.

**Table 2:** Transthoracic echocardiography parameters of both groups at first admission.

Parameters	SGLT2i Group	Control Group	*p* Value
LV end-diastolic volume (mL)	166.9 ± 62.11	155.5 ± 45.67	0.09
LVEF (%)	32.54 ± 8.98	32.00 ± 10.31	0.65
LA volume (mL)	66.31 ± 21.16	60.34 ± 15.35	0.009 **
LA diameter (cm)	4.38 ± 0.76	4.13 ± 0.58	0.003 **

Data are presented as mean ± standard deviation; ** *p* values < 0.01; LV = left ventricle; LVEF = left ventricle ejection fraction; LA = left atrium.

**Table 3:** Transthoracic echocardiography parameters of both groups at first and second admission.

Parameters	Groups	First Admission	Second Admission	*p* Value
LV end-diastolic volume (mL)	SGLT2i group	166.9 ± 62.11	162.8 ± 63.48	0.03 *
	Control group	155.5 ± 45.67	163.0 ± 49.33	0.0002 **
LVEF (%)	SGLT2i group	32.54 ± 8.98	33.85 ± 8.88	0.01 *
	Control group	32.00 ± 10.31	28.10 ± 11.15	<0.0001 **
LA volume (mL)	SGLT2i group	66.31 ± 21.16	64.04 ± 18.63	0.0003 **
	Control group	60.34 ± 15.35	68.69 ± 15.87	<0.0001 **
LA diameter (cm)	SGLT2i group	4.38 ± 0.76	4.31 ± 0.74	0.14
	Control group	4.13 ± 0.58	4.26 ± 0.59	0.02 *

Data are presented as mean ± standard deviation; * *p* values < 0.05; ** *p* values < 0.01; LV = left ventricle; LVEF = left ventricle ejection fraction; LA = left atrium.

**Table 4:** Atrial fibrillation onset in both groups.

	SGLT2i Group	Control Group	*p* Value	Odds Ratio (95% CI)
Atrial fibrillation	9 (6.25%)	28 (24.14%)	<0.0001 **	4.773 (2.150 to 10.81)

Data are presented as number and percentage; ** *p* values < 0.01.

**Table 5:** Correlation of atrial fibrillation onset and number of comorbidities in both groups.

Number of Comorbidities	SGLT2i Group	Control Group
r	0.246	0.225
95% CI	0.08 to 0.39	0.04 to 0.39
*p*	0.003 **	0.01 *

r = Pearson correlation coefficient; 95% CI = confidence interval for r; * *p* values < 0.05; ** *p* values < 0.01.

**Table 6:** Multiple linear regression analysis for predictors of new-onset atrial fibrillation.

Predictor Variable	Coefficient (β)	95% CI	Standard Error	*p*-Value
Intercept	0.155	0.010 to 0.301	0.074	0.03 *
SGLT2i treatment	−0.249	−0.338 to −0.161	0.045	<0.0001 **
Arterial hypertension	0.152	0.059 to 0.244	0.047	0.001 **
Chronic coronary syndrome	−0.142	−0.266 to −0.018	0.063	0.02 *
Type 2 diabetes	0.078	−0.005 to 0.160	0.042	0.06
Obesity	−0.089	−0.179 to 0.002	0.046	0.05
Chronic kidney disease	0.115	0.032 to 0.198	0.042	0.006 **
Smoking	0.086	−0.003 to 0.175	0.045	0.5
Obstructive sleep apnea	0.210	0.099 to 0.320	0.056	0.0002 **

95% CI = confidence interval for β; * *p* values < 0.05; ** *p* values < 0.01.

## Data Availability

Data are contained within the article.

## Abbreviations

The following abbreviations are used in this manuscript:

AF	Atrial fibrillation
HF	Heart failure
HFpEF	Heart failure with preserved ejection fraction
HFmrEF	Heart failure with mildly reduced ejection fraction
HFrEF	Heart failure with reduced ejection fraction
NYHA	New York Heart Association
SGLT2i	Sodium–glucose cotransporter 2 inhibitors
SD	Standard deviation
LV	Left ventricle
LVEF	Left ventricle ejection fraction
LA	Left atrium
